# Glaucoma: Its Symptoms, Diagnosis, and Treatment

**Published:** 1865-02

**Authors:** 


GLAUCOMA: Its Symptoms, Diagnosis, and Treatment. By PETER DIREK KEYSER, M.D. Philadelphia: LINDSAY & BLAKISTON. 1864.

This is a monograph of 88 very neatly printed pages, in paper cover. The author gives a very interesting summary of what is known in regard to causes, symptoms, and treatment of glaucoma. It is worthy of a careful perusal.

